# Non-aneurysmal perimesencephalic subarachnoid hemorrhage in a middle-aged female possibly triggered by Valsalva-like event and uncontrolled hypertension: a case report

**DOI:** 10.1097/MS9.0000000000005690

**Published:** 2026-09-09

**Authors:** Abhishek Shrestha, Diksha Shrestha, Prabin Duwadee, Saroj Gc

**Affiliations:** a Internal Medicine, Maharajgunj Medical Campus, Tribhuvan University Institute of Medicine, Maharajgunj, Kathmandu, Nepal; b Pathology, Maharajgunj Medical Campus, Tribhuvan University Institute of Medicine, Maharajgunj, Kathmandu, Nepal; c Internal Medicine, Chitwan Medical College, Bharatpur, Chitwan

**Keywords:** headache, hypertension, intracranial aneurysm, subarachnoid hemorrhage, Valsalva maneuver

## Abstract

**Introduction:**

Non-aneurysmal perimesencephalic subarachnoid hemorrhage (NAPMSAH) is a rare subtype of subarachnoid hemorrhage with a generally benign course and accounts for a small proportion of spontaneous SAH cases. Compared with other SAH populations, patients with this condition are more commonly male and less likely to have a history of smoking or hypertension.

**Case presentation:**

We report a 58-year-old hypertensive woman who presented with sudden, severe headache and repeated vomiting without focal neurological deficits. She also had a history of intermittent vomiting associated with gastroesophageal reflux disease. Brain CT showed perimesencephalic subarachnoid hemorrhage, and CT angiography was negative for aneurysm or vascular malformation, confirming NAPMSAH. She was managed conservatively, with full clinical recovery.

**Discussion:**

NAPMSAH is believed to result from venous congestion due to impaired deep cerebral venous drainage, possibly triggered by hypertension and Valsalva-like maneuvers like vomiting. Although the prognosis is excellent, aneurysmal SAH must be excluded, and imaging remains crucial for diagnosis and risk stratification.

**Conclusion:**

NAPMSAH is a rare but important cause of acute headache, with generally good outcomes. Early recognition and imaging are essential to exclude aneurysmal hemorrhage and ensure appropriate management.

## Introduction

Subarachnoid hemorrhage (SAH) is a serious type of hemorrhagic stroke, accounting for about 3–5% of all cerebrovascular diseases and affecting nearly 500 000 people worldwide each year[1]. Although traumatic SAH is more common, non-traumatic SAH is relatively rare, with a global incidence of around 6 per 100 000 individuals annually, and most cases result from ruptured saccular aneurysms and have mortality rates exceeding 50%[2]. Non-aneurysmal SAH is even less frequent, with non-aneurysmal perimesencephalic subarachnoid hemorrhage (NAPMSAH) comprising roughly 10% of spontaneous SAH cases[2]. PMSAH was first described by Jan van Gijn *et al* in 1985 as a distinct subtype of subarachnoid hemorrhage characterized by bleeding predominantly in the perimesencephalic cisterns on CT, in which a non-aneurysmal origin can be anticipated even when basal cisternal blood is present[3]. NAPMSAH has a good prognosis, with a lower incidence of complications^[^2,4^].^ Affected patients are often younger and are less commonly female, hypertensive, or smokers compared with other SAH groups^[^2,5^]^. However, identifying definitive risk factors remains difficult because of the limited number of reported cases. Although the exact cause is unknown, Valsalva-like maneuvers that cause a sudden rise in intracranial pressure, with venous congestion and rupture of the perimesencephalic veins, have been proposed[6]. We present the case of a 58-year-old hypertensive woman who presented with headache and vomiting and was later diagnosed with NAPMSAH.

This case report is in accordance with the CARE Guidelines[7]. The case report is compliant with the TITAN Guidelines 2025, which govern the declaration and use of AI[8]. Hence, no AI was used in this research paper.

HIGHLIGHTS

- Non-aneurysmal perimesencephalic subarachnoid hemorrhage (NAPMSAH) is a rare subtype of subarachnoid hemorrhage with a generally benign course.
- NAPMSAH can be a diagnostic challenge and may be missed, as patients may present without neurological deficits or signs of meningismus.
- Occurrence in a hypertensive woman is atypical, as most reported cases involve non-hypertensive men.
- Valsalva-like maneuvers, such as vomiting, along with uncontrolled hypertension, may contribute to its pathogenesis.
- Cerebral angiography is essential to rule out an aneurysmal source, as a seemingly benign PMSAH pattern may still be associated with a ruptured aneurysm and a worse prognosis.

## Case presentation

A 58-year-old woman with a known history of systemic hypertension and dyslipidemia for the past 5 years presented to the emergency department with an acute-onset headache of 1-day’s duration. The headache was initially mild but progressively worsened over time, becoming severe over the preceding 6 hours. It was localized to the frontal and parietal regions, continuous in nature, and described as the “worst headache” of her life, with an intensity of 8/10. The headache was associated with multiple episodes of vomiting (approximately 6–7), photophobia, and phonophobia.

There was no history of loss of consciousness, seizures, abnormal body movements, altered sensorium, fever, dizziness, or focal neurological deficits. She also reported a history of gastroesophageal reflux disease and recurrent episodes of dry cough, frequently associated with vomiting. She is a teacher by profession, and her job requires prolonged standing. Her past surgical history included bilateral varicose vein surgery performed 2 years earlier with a good postoperative outcome. Her medications included a fixed-dose combination of amlodipine (5 mg) and losartan (50 mg) once daily and rosuvastatin 5 mg once daily.

On arrival, her blood pressure was 160/100 mmHg, her heart rate was 69 beats per minute, and her oxygen saturation was 99% on room air. Neurological examination revealed a Glasgow Coma Scale (GCS) score of 15 (E4V5M6). Pupils were bilaterally equal (3 mm) and reactive to light. There were no signs of meningeal irritation, including neck rigidity. Cranial nerve examination was normal, with an intact gag reflex and normal swallowing. Motor examination revealed normal tone and full power (5/5) in all four limbs, with intact sensation across all dermatomes. Deep tendon reflexes were normal, and no cerebellar or gait abnormalities were noted.

### Investigations

An urgent non-contrast computed tomography (CT) scan of the head revealed subarachnoid hemorrhage involving the interpeduncular cistern, ambient cistern, quadrigeminal cistern, and bilateral superior cerebellar cisterns, consistent with a perimesencephalic pattern of hemorrhage (Fig. 1).

**Figure 1.**
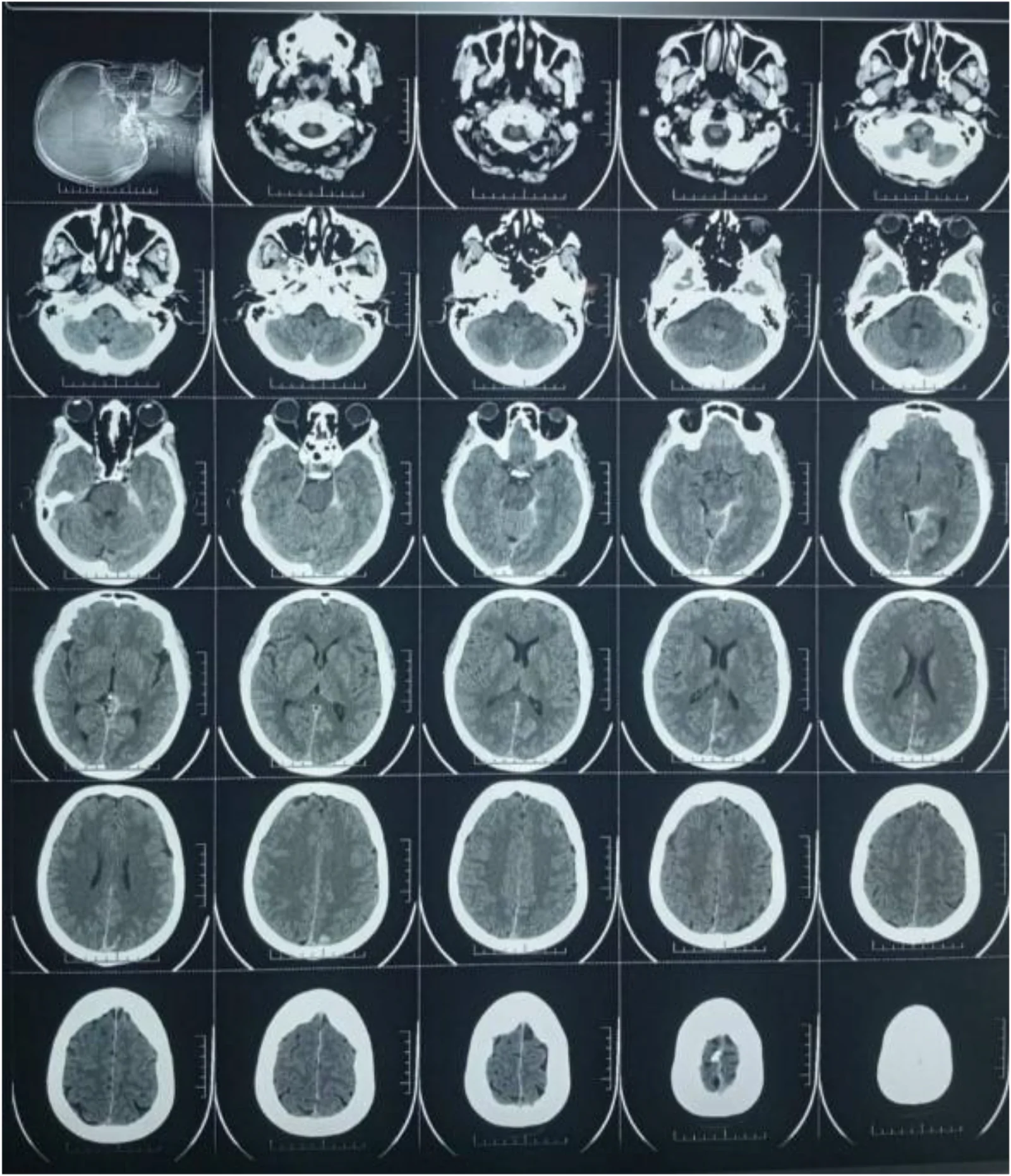
Non-contrast head CT demonstrating hyperdense subarachnoid hemorrhage predominantly centered within the perimesencephalic cisterns, consistent with a classic perimesencephalic hemorrhage pattern.

Subsequent CT cerebral angiography did not reveal any intracranial aneurysm, arteriovenous malformation, or other vascular abnormality (Fig. 2). Conventional catheter cerebral angiography [digital subtraction angiography (DSA)] was planned to definitively exclude an aneurysmal source; however, it was not performed because the patient declined due to financial constraints. Routine laboratory investigations, including complete blood count, renal function tests, liver function tests, serum electrolytes (including calcium, magnesium, and phosphorus), electrocardiogram, and routine urinalysis, were within normal limits. Serum sodium levels were monitored closely during hospitalization to evaluate for the syndrome of inappropriate antidiuretic hormone secretion (SIADH) and remained within the normal range.

**Figure 2.**
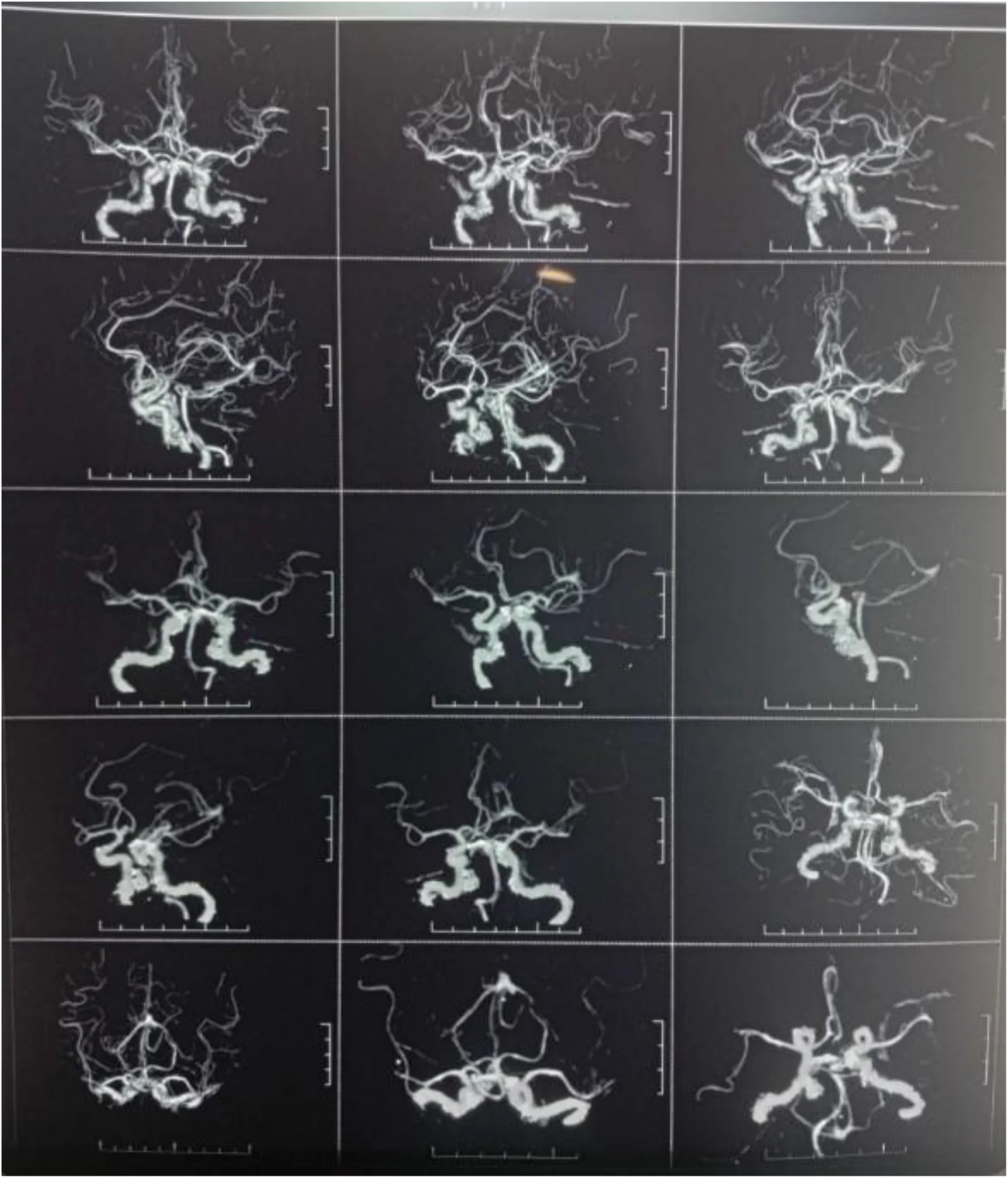
CT cerebral angiography (CTA) with three-dimensional reconstruction demonstrating normal intracranial arteries without evidence of aneurysm, arteriovenous malformation, or other vascular abnormality.

### Differential diagnosis

The initial differential for acute, severe headache with vomiting included vascular, infectious, structural, and primary headache causes. Vascular etiologies such as non-traumatic SAH, intracerebral hemorrhage, and hypertensive emergency were considered, along with meningitis or encephalitis, intracranial space-occupying lesions, idiopathic intracranial hypertension, and primary headaches like migraine.

Infection was unlikely given the absence of fever and meningeal signs and the presence of normal CSF, and structural lesions were excluded based on imaging and clinical features. Neuroimaging confirmed SAH, and CT angiography showed no vascular abnormality, supporting a diagnosis of non-aneurysmal perimesencephalic SAH.

### Management

The patient was managed in the intensive care unit with close neurological monitoring. She was started on oral nimodipine 60 mg every 4 hours to prevent cerebral vasospasm and intravenous levetiracetam 500 mg every 12 hours for seizure prophylaxis. Blood pressure was controlled with an intravenous labetalol infusion.

Supportive management included nil per os status initially, intravenous 0.9% normal saline for maintenance fluids, parenteral antiemetics, and intravenous paracetamol for analgesia. Peptic ulcer prophylaxis was also administered.

### Outcome and follow-up

The patient demonstrated gradual clinical improvement over 2 days in the intensive care unit and was subsequently transferred to the general ward. She remained neurologically intact throughout her hospital stay, with no complications such as vasospasm, rebleeding, or electrolyte disturbances.

She was discharged on the fifth day of hospitalization in stable condition. At follow-up visits 2 weeks and 1 month post-discharge, she reported complete resolution of headache and had no residual neurological deficits. She resumed her normal daily activities without any functional impairment.

## Discussion

NAPMSAH is a rare condition first described by van Gijn *et al* in 1985[3]. It represents a distinct subtype of spontaneous subarachnoid hemorrhage with a favorable clinical course and accounts for approximately 10% of spontaneous SAH cases^[^1,2^]^. Patients are more commonly male and are less likely to have hypertension or a history of smoking than those with aneurysmal SAH^[^2,5^]^. Although the exact pathophysiology remains uncertain, the prevailing hypothesis suggests a venous source of hemorrhage. Yamakawa *et al* proposed that anatomical variations in the deep cerebral venous drainage system, together with Valsalva-like maneuvers, may transiently increase intracranial venous pressure, resulting in congestion and rupture of fragile perimesencephalic veins[6]. Valsalva maneuvers are known to transiently elevate intracranial pressure[9], and repeated vomiting is considered a Valsalva-like event[10]. In our patient, recurrent vomiting preceded the onset of symptoms. While systemic arterial hypertension alone does not directly explain venous rupture, an acute hypertensive surge may have acted synergistically with vomiting-induced transient impairment of venous return, thereby increasing intracranial venous pressure and predisposing to hemorrhage in the presence of susceptible venous anatomy. This mechanism remains speculative but provides a physiologically plausible explanation for the clinical presentation observed in our patient^[^6,9,10^]^.

Patients with NAPMSAH usually present with sudden severe headache and meningeal symptoms like neck stiffness, photophobia, nausea, and vomiting, while loss of consciousness and focal deficits are rare[2]. Genetic susceptibility to hypertension is associated with an earlier onset of nontraumatic SAH in women, highlighting the importance of strict blood pressure control to reduce the risk and morbidity[11]. In our case, the lack of neck rigidity and neurological deficits initially suggested a primary headache, which could have led to a missed diagnosis.

Management of PMSAH is mainly conservative, emphasizing hemodynamic stabilization, complication prevention, and symptomatic treatment. Nimodipine is often used to reduce the risk of vasospasm, though its use is less common than in aneurysmal SAH[2]. Our patient showed complete clinical recovery with conservative management.

Although a classic perimesencephalic hemorrhage pattern is strongly associated with a non-aneurysmal etiology, a small proportion of patients may still harbor an aneurysm. A recent study reported that approximately 6.2% of patients with an apparently benign perimesencephalic hemorrhage pattern had a ruptured aneurysm, with 3.7% detected only by conventional catheter angiography[12]. Therefore, digital subtraction angiography (DSA) may still be considered in selected patients, particularly when the hemorrhage distribution is atypical, CT angiography quality is suboptimal, or clinical suspicion remains high despite negative CTA findings. Conversely, several contemporary studies suggest that patients with a classic perimesencephalic hemorrhage pattern and a high-quality negative CTA have an extremely low likelihood of an underlying aneurysm, and routine additional angiography is often unnecessary[13]. In our patient, DSA was planned for definitive exclusion of an aneurysmal source but was not performed because the patient declined it due to financial constraints. The patient remained neurologically stable throughout hospitalization and demonstrated complete clinical recovery on follow-up, further supporting the diagnosis of NAPMSAH.

If early CT shows a classic perimesencephalic pattern with a negative high-quality CT cerebral angiography, further imaging is usually unnecessary. Complications are rare and generally self-limiting, although hydrocephalus may occasionally require lumbar puncture. Anticoagulation should be avoided initially due to the risk of rebleeding, and the overall prognosis is excellent compared to aneurysmal SAH[13]. Less intensive monitoring and shorter hospital stays are required for PMSAH patients without aneurysm or hydrocephalus[12].

## Conclusion

An acute, severe headache should always prompt evaluation for intracranial hemorrhage, even in the absence of focal neurological deficits or meningeal signs. Although the precise pathophysiology remains incompletely understood, transient elevations in intracranial venous pressure during Valsalva-like maneuvers may contribute to NAPMSAH. At the same time, uncontrolled hypertension may act as a facilitating cofactor rather than an independent cause. Careful neurovascular imaging remains essential to exclude an aneurysmal source before establishing the diagnosis of NAPMSAH.

## Data Availability

Not applicable.

## References

[1] Mortazavi ZS Zandifar A Du J. Re-evaluating risk factors, incidence, and outcome of aneurysmal and non-aneurysmal subarachnoid hemorrhage. World Neurosurg 2023;175:e492–e504. 10.1016/j.wneu.2023.03.131 37024083

[2] Roman-filip I Morosanu V Bajko Z. Non-aneurysmal perimesencephalic subarachnoid hemorrhage: a literature review. Diagnostics (Basel) 2023;13:1195. 10.3390/diagnostics13061195 PMC10047780 36980503

[3] van Gijn J van Dongen KJ Vermeulen M. Perimesencephalic hemorrhage: a nonaneurysmal and benign form of subarachnoid hemorrhage. Neurology 1985;35:493–97. 10.1212/wnl.35.4.493 3982634

[4] Kim M Kim BJ Son W. Retrospective 6-month outcomes and prognostic factors following spontaneous angiogram-negative non-perimesencephalic subarachnoid hemorrhage. J Clin Neurosci 2022;96:12–18. 10.1016/j.jocn.2021.12.017 34959170

[5] Koller EC Steynberg JC Jooma Z. Perimesencephalic subarachnoid haemorrhage: a case study. J Coll Med South Afr 2025;3. doi:10.4102/jcmsa.v3i1.245 PMC12505818 41070174

[6] Yamakawa H Ohe N Yano H. Venous drainage patterns in perimesencephalic nonaneurysmal subarachnoid hemorrhage. Clin Neurol Neurosurg 2008;110:587–91. 10.1016/j.clineuro.2008.03.001 18433987

[7] CARE Case Report Guidelines. CARE Checklist. Accessed 29 July 2026. https://www.care-statement.org/checklist.

[8] Agha R Mathew G Rashid R. Transparency in the Reporting of Artificial Intelligence (TITAN) guideline. Prem J Sci 2025. doi:10.70389/pjs.100082

[9] Samuel, Yiangou A Thaller M Thaller M. The impact of valsalva manoeuvres and exercise on intracranial pressure and cerebrovascular dynamics in idiopathic intracranial hypertension. Neuro-Ophthalmology 2024;48:122–33. 10.1080/01658107.2023.2281433 PMC10936629 38487358

[10] Tiecks FP Lam AM Matta BF. Effects of the valsalva maneuver on cerebral circulation in healthy adults. Stroke 1995;26:1386–92. 10.1161/01.str.26.8.1386 7631342

[11] Vanent KN Leslie-mazwi TM Matouk CC. Hypertension is associated with earlier onset of nontraumatic subarachnoid hemorrhage in women: a mendelian randomization study. Stroke 2025;56:2996–3001. 10.1161/STROKEAHA.124.047631 40698383

[12] Alrohimi A Davison MA Pandhi A. Aneurysmal versus “benign” perimesencephalic subarachnoid hemorrhage. Stroke Vasc Interv Neurol 2024;4. doi:10.1161/SVIN.123.001166 PMC12778503 41584481

[13] Mensing LA Vergouwen MDI Laban KG. Perimesencephalic hemorrhage. Stroke 2018;49:1363–70. 10.1161/STROKEAHA.117.019843 29695465

